# Enhancing cancer stage prediction through hybrid deep neural networks: a comparative study

**DOI:** 10.3389/fdata.2024.1359703

**Published:** 2024-03-22

**Authors:** Alina Amanzholova, Aysun Coşkun

**Affiliations:** ^1^Graduate School of Natural and Applied Sciences, Department of Computer Engineering, Gazi University, Ankara, Türkiye; ^2^Khoja Akhmet Yassawi International Kazakh-Turkish University, Faculty of Engineering, Department of Computer Engineering, Turkistan, Kazakhstan; ^3^Department of Computer Engineering, Faculty of Technology, Gazi University, Ankara, Türkiye

**Keywords:** cancer stage prediction, artificial intelligence, deep belief network, mRNA expression, DNA methylation

## Abstract

Efficiently detecting and treating cancer at an early stage is crucial to improve the overall treatment process and mitigate the risk of disease progression. In the realm of research, the utilization of artificial intelligence technologies holds significant promise for enhancing advanced cancer diagnosis. Nonetheless, a notable hurdle arises when striving for precise cancer-stage diagnoses through the analysis of gene sets. Issues such as limited sample volumes, data dispersion, overfitting, and the use of linear classifiers with simple parameters hinder prediction performance. This study introduces an innovative approach for predicting early and late-stage cancers by integrating hybrid deep neural networks. A deep neural network classifier, developed using the open-source TensorFlow library and Keras network, incorporates a novel method that combines genetic algorithms, Extreme Learning Machines (ELM), and Deep Belief Networks (DBN). Specifically, two evolutionary techniques, DBN-ELM-BP and DBN-ELM-ELM, are proposed and evaluated using data from The Cancer Genome Atlas (TCGA), encompassing mRNA expression, miRNA levels, DNA methylation, and clinical information. The models demonstrate outstanding prediction accuracy (89.35%−98.75%) in distinguishing between early- and late-stage cancers. Comparative analysis against existing methods in the literature using the same cancer dataset reveals the superiority of the proposed hybrid method, highlighting its enhanced accuracy in cancer stage prediction.

## 1 Introduction

The timely identification and effective treatment of cancer are paramount for enhancing patient outcomes and curbing disease progression (Mohtasebi et al., 2023). As the landscape of cancer prediction evolves, artificial intelligence (AI) technologies have emerged as powerful tools to streamline this process (Monjezi et al., 2023; Morteza et al., 2023; Rezaei et al., 2023; Zeinali-Rafsanjani et al., 2023). However, challenges persist, particularly in accurately categorizing cancer stages based on gene sets. Issues such as limited sample sizes, data dispersion, and the use of linear classifiers with simplistic parameters have impeded the progress of prediction algorithms.

The field of cancer diagnosis and prediction using artificial intelligence (AI) methods has seen significant growth in recent years (Rezaei et al., 2022). AI techniques, such as machine learning (ML) and deep learning (DL), have been increasingly applied in oncology for various purposes, including the detection and diagnosis of cancer (Chugh et al., 2021; Painuli and Bhardwaj, 2022; Rana and Bhushan, 2023). The application of AI in oncology is not only limited to clinical practice but also extends to research fields with contributions from medicine, computer science, mathematics, and engineering (Murthy and Bethala, 2023). AI methods have shown promise in increasing diagnostic accuracy and efficiency by providing quantifiable outputs to predict cancer behavior and prognosis (Choupanzadeh and Zadehgol, 2023). For instance, ML has been shown to reduce variability in grading dysplasia and cancers, ensuring standardization and consistency, which is crucial for informing treatment decisions (Yadavendra and Chand, 2020; Shaikh and Rao, 2022; Huang et al., 2023). The use of computational methods to learn information directly from data, whether through supervised or unsupervised learning, has been a significant development in the field (Sultan et al., 2020; Castiglioni et al., 2021; Jafarzadeh Ghoushchi et al., 2023). AI-based image analysis from whole slide images of human tissue has demonstrated potential in reliably predicting diagnosis, prognosis, mutational status, and response to treatment in various cancers, including colorectal, lung, skin, and breast malignancies (Naseem et al., 2022).

A fully connected deep neural network was developed by Ahn et al. (2018) from 6,703 tumors and 6,402 normal samples, and the contribution of individual genes was evaluated. In a similar effort, Li et al. (2017, 2021) classified individual tumor types. By coupling a k-nearest neighbor algorithm with a genetic algorithm for gene selection, Lyu and Haque (2018) were able to predict 31 types of cancer with a high level of accuracy. For each of the 33 TCGA cancer types analyzed, Selvaraju et al. (2017) achieved more than 95% accuracy by using a CNN model with 2D mapping of gene expression samples as inputs. Additionally, Liang et al. (2020) developed a method of interpreting data using the guided grad-CAM to identify the facial features of cancer patients. Based on GeneCT (Sun et al., 2018), input genes are divided into two categories: oncogenes and tumor suppressors, which allow identification of cancer status, and transcription factors, which allow identification of tissue origin. In this context, tissue origin refers to the specific organ or tissue type from which the cancer has originated. The identification of transcription factors associated with input genes aids in discerning the tissue or organ where the cancer has initiated. This information is valuable for understanding the specific anatomical site affected by the cancerous condition. Yuan et al. (2018) delved into cancer-type prediction using copy number aberration and chromatin 3D structure, employing convolutional neural networks (CNNs). Their investigation, as reflected in experimental outcomes on the COSMIC CNA dataset (Forbes et al., 2017), highlights the optimal performance achieved by a 2D CNN utilizing both cell lines of HiC data. The output of the model corresponds to the total number of cancer types, which in this instance is 25, resulting in an impressive accuracy rate of 61%. To a certain extent, all of these attempts were accurate; however, these methods do not take into account the possibility of tissue of origin within each type of cancer. A data interpretation scheme that has not been designed to remove the effect of normal tissues during cancer arrangement will not be able to differentiate between tissues or types of cancer in the absence of removing these influences. This makes such models ineffective for the analysis of functional data or the selection of biomarkers for cancer detection. There was also no systematic examination of the effect of different CNN model constructions on prediction accuracy in any of these studies. Ma et al. (2020) introduced a prediction model based on extreme gradient boosting to distinguish early-stage from late-stage cancers. In the context of predicting the stage of breast cancer, they employed the extreme gradient boosting method. The average prediction accuracy scores for the four cancers were 0.808 for KIRC, 0.872 for KIRP, 0.600 for HNSC, and 0.595 for LUSC. It's crucial to highlight that despite these accuracy scores, the extreme gradient boosting method does have a drawback, namely, the potential for low accuracy. This limitation increases the risk of errors in the prediction process, necessitating careful consideration of its application in clinical contexts.

In addition to conducting a comprehensive statistical analysis, the integration of machine learning algorithms holds promise in identifying key driving mutations. Gene expression data is a widely utilized data type for cancer prediction in numerous studies (Nguyen and Rocke, 2002; Xiao et al., 2018; Huang et al., 2021). However, a significant challenge arises from the small sample size coupled with high dimensionality. While each sample may contain thousands of genes, only a subset is pertinent to the target disease, rendering most genes irrelevant (Wang et al., 2008). To address the high dimensionality issue, gene selection methods are commonly employed before prediction (Mostavi et al., 2020; Mazlan et al., 2021; Varnier et al., 2021). Nevertheless, this step may inadvertently discard genes that, while having minor effects on disease generation in general, remain significant for diagnosing specific cancer types in certain patients. Furthermore, the inclusion of irrelevant genes introduces noise and diminishes the performance of machine-learning classifiers (Yang et al., 2018). Despite these advances, the integration of AI into clinical practice faces several challenges, including the need for large, multicenter clinical trials to validate AI systems in real-time clinical settings (Abbasi et al., 2020; Alhasan, 2021; Talukder et al., 2022). The potential of AI to improve the quality of care in healthcare systems is significant, as AI-based risk prediction models can investigate complex relationships between clinical data and disease treatment. In summary, AI methods are transforming cancer diagnosis and prediction, offering tools for more consistent, efficient, and accurate diagnosis, which can aid clinical decision-making and potentially improve patient survival. However, further research and development are needed to overcome current limitations and fully realize the potential of AI in oncology.

According to the literature, the accurate prediction of cancer stages based on gene sets presents notable challenges. Issues such as limited sample volumes, data dispersion, and the use of linear classifiers with simplistic parameters impede the improvement of prediction performance. This paper addresses these challenges by proposing a novel approach that leverages the power of hybrid deep neural networks for the prediction of early and late-stage cancers. The integration of Genetic Algorithms (GA), Extreme Learning Machines (ELM), and Deep Belief Networks (DBN) forms the foundation of our innovative method. Employing a deep neural network classifier developed with the Tensorflow framework and Keras libraries, our study aims to enhance the accuracy of cancer stage prediction. To assess the efficacy of our proposed method, we conducted extensive evaluations using data sourced from The Cancer Genome Atlas (TCGA), encompassing diverse information such as mRNA expression levels, miRNA levels, DNA methylation data, and clinical information. Two distinct evolutionary techniques, namely DBN-ELM-BP and DBN-ELM-ELM, are introduced and rigorously tested. Our findings reveal exceptional prediction accuracy in distinguishing between early- and late-stage cancers, demonstrating the potential of our hybrid model. Furthermore, we conduct a comparative analysis against state-of-the-art methods in the literature, affirming the superiority of our proposed hybrid method in cancer stage prediction. This research contributes to the ongoing efforts to improve cancer diagnosis and treatment, offering a promising avenue.

## 2 Materials and methods

This study introduces an innovative method for forecasting early and late-stage cancers by integrating hybrid deep neural networks. In general, “early-stage” cancers are characterized by localized growth, confined to the site of origin and limited spread, often corresponding to lower numerical stages (e.g., Stage I or Stage 0). In contrast, “late-stage” cancers have progressed beyond the initial site, involving invasion of nearby tissues or metastasis, and are associated with higher numerical stages (e.g., Stage III or Stage IV).

**1) Early-Stage Cancers:**

- Early-stage cancers are characterized by localized growth, meaning that the tumor is confined to its site of origin and has not yet invaded neighboring tissues or spread to distant organs.

- In the context of cancer staging, early-stage cancers are typically associated with lower numerical stages (e.g., Stage I or Stage 0), indicating a smaller tumor size and limited spread.

**2) Late-Stage Cancers:**

- Late-stage cancers, on the other hand, have advanced beyond the initial site of origin and often involve the invasion of nearby tissues or the spreading (metastasis) to distant parts of the body.

- Higher numerical stages (e.g., Stage III or Stage IV) are indicative of late-stage cancers, signifying a more extensive disease with greater tumor size and potential involvement of lymph nodes or distant organs.

The distinction between early and late stages is crucial in cancer diagnosis and treatment planning. Early detection of cancer allows for more effective and less aggressive treatment options, often resulting in better outcomes for patients. Late-stage cancers, with their increased complexity and potential for metastasis, often require more aggressive interventions and may have a poorer prognosis.

### 2.1 Dataset

**Figure 1:**
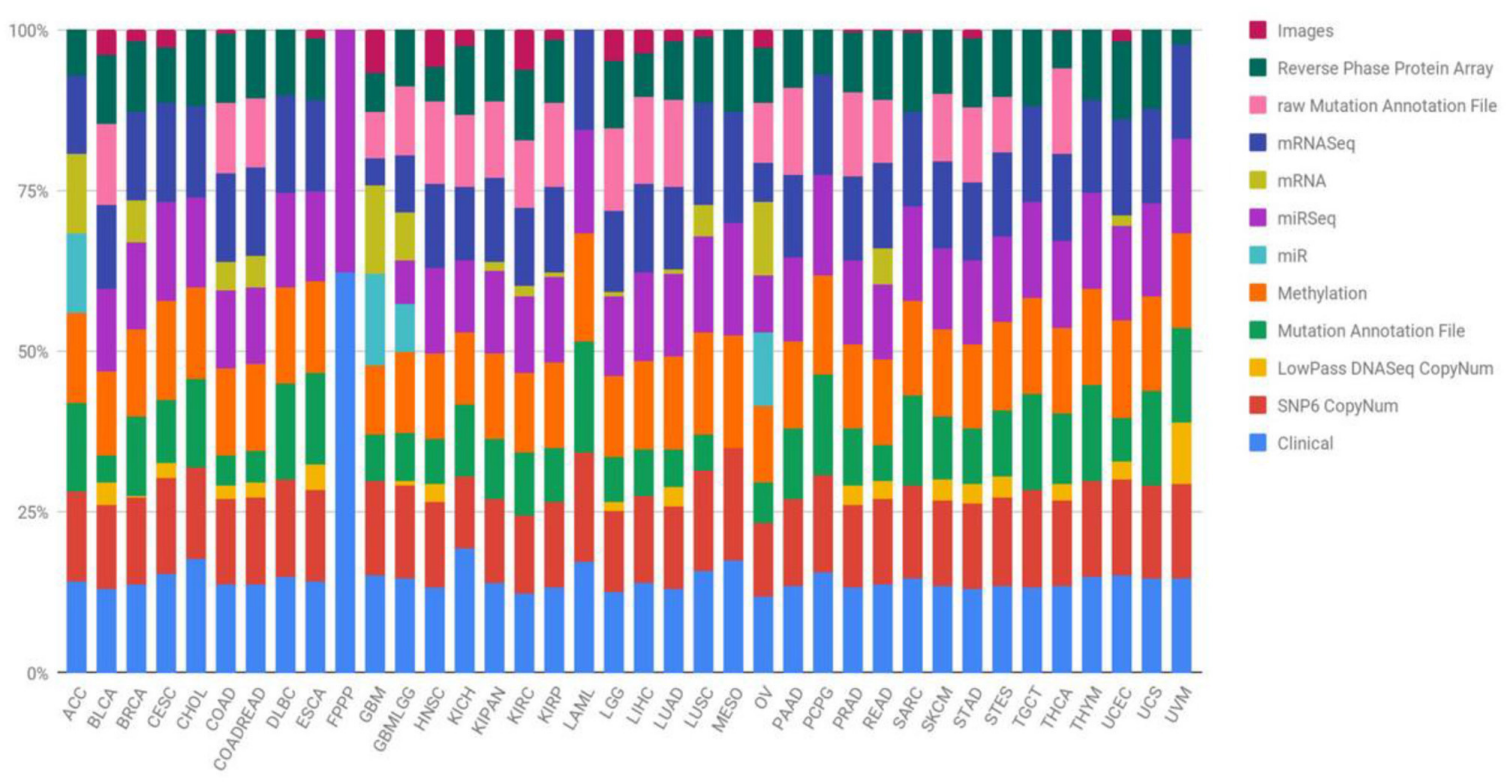
Quantification of TCGA repository samples by tumor type and biotechnological analysis (Liñares-Blanco et al., 2021).

This study utilized data sourced from TCGA, specifically focusing on four prominent cancer types (Wang et al., 2016). In 2006, an initial pilot initiative demonstrated the feasibility of creating a specific atlas detailing genetic changes unique to various cancer types. Subsequently, the TCGA dataset has expanded its efforts, amassing tissues from over 11,000 patients, encompassing more than 33 types and subtypes of cancer, including 10 rare forms of cancer. A noteworthy aspect of this undertaking is the unrestricted availability of all collected information to any researcher interested in directing their investigations toward these diseases. Figure 1 succinctly outlines the diverse types of data provided by the TCGA project and visually represents the percentage contribution of each data type to the overall subtype. Quantifying the number of samples within the TCGA repository involves categorizing them based on both the type of tumor and the specific biotechnological analysis employed. The open-access nature of the data streamlines the development of innovative models, eliminating the need for an initial financial investment to acquire the necessary information.

**Figure 2:**
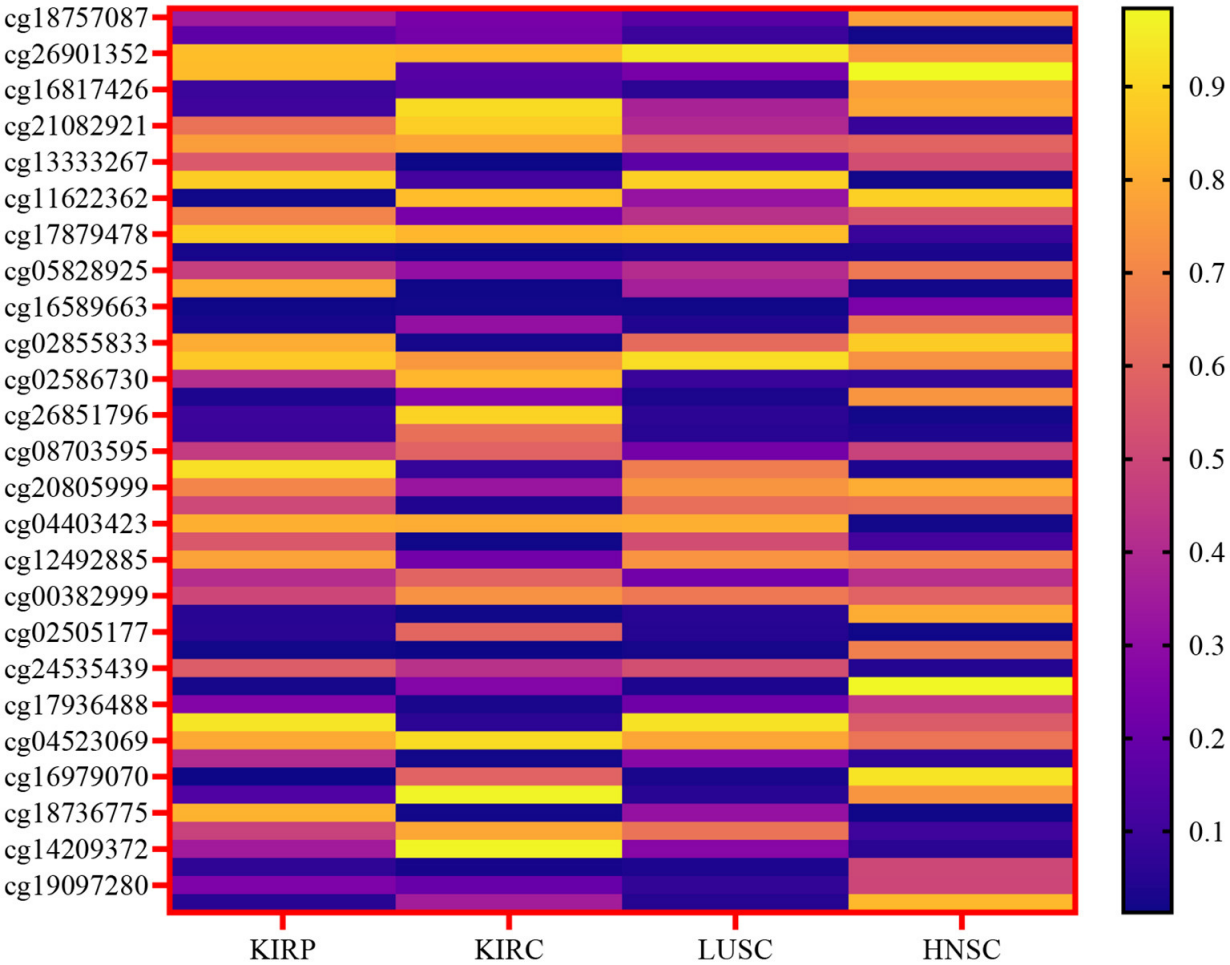
Heatmap of the expression levels of DNA methylation hyperparameter for a selected gene across TCGA samples, specifically focusing on the KIRP, KIRC, LUSC, and HNSC cancer types.

The primary emphasis was on the top four types of cancer, and our data collection encompassed 1,392 patients diagnosed with kidney cancer. The kidney cancer subtypes included were kidney renal papillary cell carcinoma (KIRP) and kidney renal clear cell carcinoma (KIRC), along with lung squamous cell carcinoma (LUSC) and head and neck squamous cell carcinoma (HNSC). The present study focused on four types of biotechnological data, namely miRNA-seq data, DNA methylation data, cancer stage, and mRNA expression. In the TCGA dataset, these features are represented numerically for various genes, and the respective ranges for each quantity are detailed in Table 1. Figure 2 depicts a heatmap showcasing the expression levels of DNA methylation hyperparameter for 50 chosen genes across TCGA samples, with a specific emphasis on the KIRP, KIRC, LUSC, and HNSC cancer types.

**Table 1:** Range of DNA methylation, miRNA-seq, and mRNA hyperparameters.

	**KIRP**	**KIRC**	**LUSC**	**HNSC**
DNA methylation	0.05–1.01	0.05–1.02	0.01–1.02	0.01–0.99
miRNA-seq	800–15,000	200–16,000	400–15,000	500–14,000
mRNA	0.2–120	0.1–180	0.5–150	0.1–200
Cancer stage	I or II	I or II	I or II	I or II

In this study, we recognize the significance of considering the unique molecular characteristics and clinical behaviors associated with different types of cancers. Therefore, to ensure the specificity of our classification models, we adopted a meticulous approach by training separate neural networks for each of the four cancer types under investigation—kidney renal papillary cell carcinoma (KIRP), kidney renal clear cell carcinoma (KIRC), lung squamous cell carcinoma (LUSC), and head and neck squamous cell carcinoma (HNSC). This individualized training strategy allows us to tailor the models to the distinct features of each cancer type, thereby enhancing the accuracy and reliability of our results.

### 2.2 Data pre-processing

The primary objective of data pre-processing and cleaning is to enhance data quality, especially considering the inevitability of missing data in medical research. Our analysis involves scrutinizing a patient's DNA methylation, mRNA, and microRNA levels for each cancer type (Table 2). Within our dataset, certain features exhibit missing values. In adherence to prior research practices, any patient information with even a single missing data item was traditionally discarded—a practice we aim to continue. Therefore, it becomes imperative to address these missing values by imputing appropriate data before modeling.

**Table 2:** KIRC, KIRP, LUSC, and HNSC datasets (Ma et al., 2020).

**Cancer type**	**Class**	**Number**	**DNA methylation**	**mRNA**	**microRNA**
KIRC	Early	175	16,335	16,387	385
	Late	120			
KIRP	Early	195	16,740	16,469	350
	Late	55			
LUSC	Early	294	16,586	16,598	417
	Late	53			
HNSC	Early	107	16,598	16,207	442
	Late	386			

In this work, we harnessed the power of multiple types of data to gain a comprehensive understanding of the molecular landscape associated with different cancer types. Specifically, we integrated microRNA data, cancer stage information, DNA methylation data, and mRNA expression data. Each of these data types offers unique insights into the molecular characteristics of cancer, capturing different aspects of its complexity.

mRNA-seq data provides valuable information about the regulatory role of microRNAs, while cancer stage information allows us to contextualize molecular alterations in the progression of the disease. DNA methylation data offers insights into epigenetic modifications that can influence gene expression, and mRNA expression data provides a snapshot of active genes in the cellular environment. By integrating these diverse datasets, our approach aims to unravel the intricate molecular details of cancer, identify potential biomarkers, elucidate regulatory networks, and explore the clinical implications of molecular alterations at various stages of the disease.

This integrative strategy is grounded in the fundamental principles of cancer biology, recognizing that a holistic view of the molecular landscape is crucial for a comprehensive understanding of the disease. By exploring the relationships among different data layers, we can unveil hidden patterns, discover novel associations, and contribute to advancing our knowledge of cancer biology.

The Expectation-Maximization algorithm (Ng et al., 2012) serves as a method for estimating maximum likelihood in scenarios involving latent variables. In the realm of machine learning algorithms, this method proves to be a versatile and effective approach, contingent on the inclusion of all relevant interacting random variables within the training dataset. However, when latent variables—unobserved or hidden variables that interrelate with those in the dataset—are introduced, the maximum likelihood estimation becomes challenging. In situations where data is missing, the Expectation-Maximization algorithm stands out as an efficient iterative process for computing the maximum likelihood estimate. The algorithm comprises two main stages in each iteration: the expectation stage and the maximization stage. The expectation stage involves estimating the values of latent variables, while the maximization stage entails optimizing the parameters of the model based on the observed and estimated latent variables. Through repeated iterations, with an assurance that the likelihood value increases at each step, the algorithm converges to a stable solution (Schön, 2009; Gupta and Chen, 2011).

For normalization, the minimum-maximum technique was employed as one of the pre-processing steps, as described in Eq. 1.


(1) $$\begin{array}{l} Y_{new}=2\frac{y-y_{\min}}{y_{\min}-y_{\max}}-1 \end{array}$$

where *y*_min_ and *y*_max_ are the minimum and maximum of the parameters.

### 2.3 Proposed models

Stage I was labeled early-stage, while stage II was labeled late-stage literature (Rahimi and Gönen, 2018). Samples from each cancer type were randomly allocated to three distinct datasets as follows: (1) a training set comprising 70% of the samples, (2) a validation set encompassing 10% of the samples, and (3) a testing set containing 20% of the samples. This division ensures a representative distribution of data across these sets, facilitating robust model training, validation, and evaluation. The training dataset served as the foundation for model training, while the test dataset was employed for evaluating the model performance. To address the imbalance present within the training dataset, oversampling was implemented concerning the target variable. This approach aims to ensure a more equitable representation of different classes, enhancing the model's ability to generalize and make accurate predictions. In the framework of this study, both the testing and training datasets are randomly selected from the main datasets for each neural network. The development of a deep neural network classifier is implemented using the TensorFlow framework and the Keras libraries.

#### 2.3.1 Deep belief network

A DBN is constructed by combining several layers of the Restricted Boltzmann Machine (RBM). Unsupervised learning is performed in one visible layer and one hidden layer of an RBM (Sohn, 2021). Essentially, the data are divided into two layers: a visible layer and a hidden layer. Except for the hidden layer of the last RBM in a DBN, the hidden layer of each RBM in a DBN is treated as the visible layer of the next RBM. Based on RBMs, the probability distribution of visible variables is calculated using the hidden layer (Hinton, 2009).

#### 2.3.2 Evolving DBN weights

Typically, the backpropagation (BP) algorithm is a feed-forward supervised neural network training algorithm used to fine-tune deep belief networks' weights (Rumelhart et al., 1995). In this algorithm, there is a risk of becoming trapped in the local minimum of the error function when learning patterns among data. The global minimum cannot be found by BP when the error function is multivariate or non-differentiable. The best set of network weights can be found by using evolutionary techniques. A genetic algorithm searches for optimal or near-optimal solutions to different types of objective functions using an evolutionary algorithm.

In WE-DBN, the optimization of network weights occurs through a genetic algorithm tailored to a predefined architecture. The acronym “WE” denotes the evolution of weights within the deep belief network facilitated by genetic algorithms. This model incorporates pre-trained deep belief networks, constituting chromosomes within the initial population. In the realm of optimization problems, this approach serves as a fitting initial step for the application of genetic algorithms.

#### 2.3.3 DBN-based ELM classifier

The Extreme Learning Machine (ELM) stands out as an efficient algorithm grounded in feed-forward neural networks featuring a hidden layer. Notably, ELM exhibits higher scalability, reduced computational complexity, and superior generalization performance when compared to the Backpropagation (BP) algorithm. In this study, we leverage the advantages of ELM to introduce a novel combination with a deep belief network. Unlike traditional approaches, the hidden layer in ELM does not require explicit adjustment. The connection weights between the input and hidden layers, alongside biases and hidden neurons, are generated randomly. Meanwhile, the connection weights between the hidden layer and the output layer are computed. Notably, improved results are achieved by utilizing more appropriate weights for the hidden layer, as opposed to random weights. This paper introduces a hybrid model, DBN-ELM, representing both an enhancement over the traditional DBN and an extension of the capabilities of ELM.

**Figure 3:**
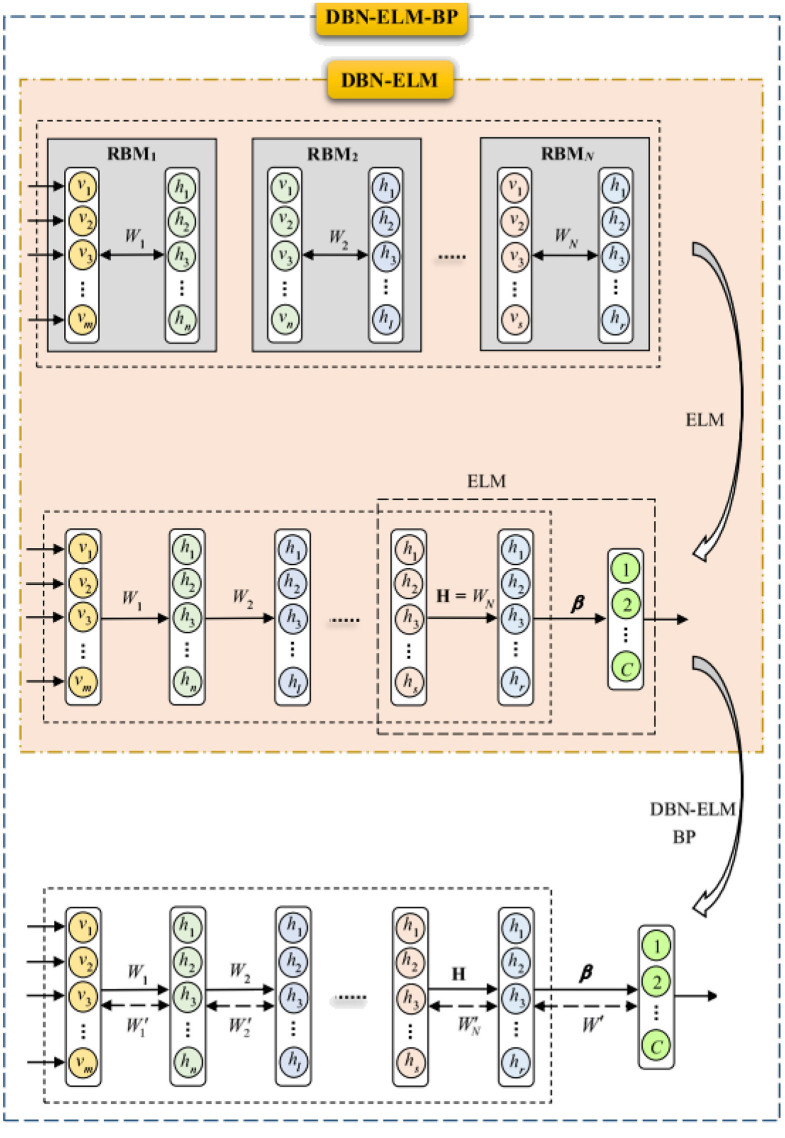
Structure of DBN-ELM and DBN-ELM-BP.

The ELM-DBN deep learning algorithm aims to procedure an extreme learning machine classifier after pertaining the network by DBN to accurately adjust the weights between the last DBN layer and the output layer (β). This model replaces the BP algorithm with an ELM classifier. The DBN-ELM model utilizes basic ELM for its advantages, such as high learning speed and good generalization performance. The selection of basic ELM was also motivated by the fact that this type of ELM is very suitable for the scale of the data sets used in this study. The inner part of Figure 3 illustrates the combination of DBN and ELM graphically. It is considered that the input and hidden layers of the last restricted Boltzmann machine and the matrix of weights between them (*W*_*N*_, a real value matrix with dimensions of *m* × *n*) are the input layer and the hidden layer of ELM, respectively.

#### 2.3.4 DBN-ELM followed by BP algorithm

The BP algorithm, known for its local search characteristic, benefits from the use of non-random and more appropriate weights, leading to faster convergence and improved prediction performance. However, in the context of deep belief network pre-training, only the last hidden layer undergoes training, leaving the weights between the final hidden layer and the output layer randomly selected. To address this limitation, the DBN-ELM-BP model incorporates an ELM classifier. This model involves a BP-supervised ELM step following unsupervised network pre-training and supervised prediction. Figure 3 visually illustrates the training process of this model.

In the initial phase, a pre-training session occurs without DBN supervision. The **H** matrix, equivalent to the weight matrix derived from the last restricted Boltzmann machine in the DBN (first fine-tuning step), is calculated using the ELM classifier for weights between the last hidden layer and output. Subsequently, after error calculation, the network's weight matrix is updated (represented by the dashed arrows), marking the second fine-tuning stage. The dashed arrow in Figure 3 denotes that the weight matrix has been updated through the BP algorithm. Notably, during the optimization of the DBN architecture, a genetic algorithm was employed for the first time in this work.

Displayed in Table 3 are the parameters governing the genetic algorithm for the evolutionary models. Determining these parameters involves a meticulous trial-and-error approach, where the values are fine-tuned to optimize the performance of the evolutionary models. This iterative process ensures that the genetic algorithm is configured with settings that enhance its efficiency and effectiveness in achieving the desired outcomes within the context of the evolutionary models. Table 3 outlines the specific parameters employed for the genetic algorithm in the evolutionary models. These parameters play a crucial role in guiding the optimization process within the context of evolutionary models, influencing their performance and outcomes. The table serves as a reference for understanding the key settings that have been configured to fine-tune the GA, ensuring its effectiveness in achieving the desired objectives within the evolutionary models.

**Table 3:** The specific parameters employed for the genetic algorithm in the evolutionary models.

**Parameter**	**Value**
Population type	Binary
Population size	50
Iteration no.	2,000
Selection method	Ranking
Crossover fraction	0.75000
Mutation probability	0.350
Crossover probability	0.9
Crossover	PMX

### 2.4 Evaluation metrics

To evaluate and determine the fitness value of each chromosome, we calculate the accuracy, sensitivity, and specificity using the training data. Calculation of the prediction accuracy percentage, sensitivity, specificity, and F1-score are based on the confusion matrix elements as shown in Eqs. 2–5.


(2) $$\begin{array}{l} \text{ACC}=\frac{TN+TP}{TN+TP+FP+FN} \end{array}$$

(3) $$\begin{array}{l} \text{SEN}=\frac{TP}{FN+TP} \end{array}$$

(4) $$\begin{array}{l} \text{SPE}=\frac{TN}{FP+TN} \end{array}$$

(5) $$\begin{array}{l} \text{F1-score}=\frac{TP}{TP+\left(FN+FP\right)/2} \end{array}$$

in which ACC, SEN, and SPE are accuracy, sensitivity, and specificity parameters, respectively. TP, TN, FP, and FN respectively denote the number of true positives, true negatives, false positives, and the number of false negatives. As a result, high sensitivity and specificity will result in higher accuracy. Furthermore, an evaluation of performance is conducted using the confusion matrix.

## 3 Results

**Figure 4:**
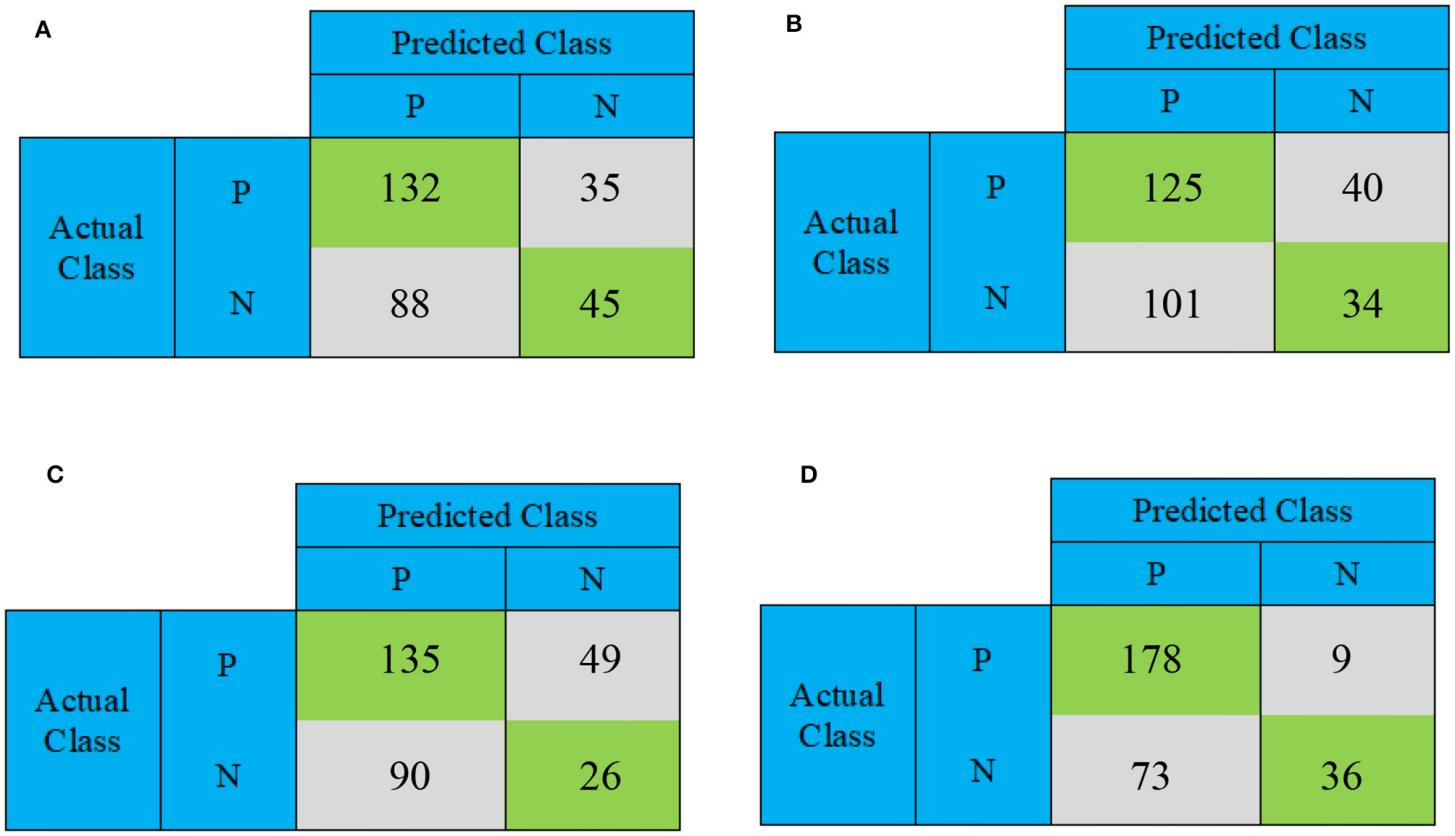
The confusion matrix for binary prediction obtained from various CNN approaches for the KIRC cancer type. The subfigures depict different models: **(A)** DBN, **(B)** WE-DBN, **(C)** DBN-ELM, and **(D)** DBN-ELM-BP.

This section conducts a comparative analysis of the results obtained from the presented DBN, WE-DBN, DBN-ELM, and DBN-ELM-BP models against various existing sequential prediction models, including the standard deep neural network. Figure 4 provides a visualization of the confusion matrix analysis for each model, focusing on the KIRC cancer type. In Figure 4, the confusion matrix for the test data of the KIRC cancer type is depicted. Notably, the DBN model correctly predicts 45 test data instances with negative labels. In comparison, the WE-DBN model achieves 34 correct predictions, the DBN-ELM model attains 26 correct predictions, and the DBN-ELM-BP model achieves 36 correct predictions. These results offer insights into the performance variations among the different models in the context of KIRC cancer-type prediction. Similarly, 132 positively labeled data can be correctly predicted by the DBN model, whereas the WE-DBN model can predict 125 data, the DBN-ELM model can predict 135 data, and the DBN-ELM-BP model can predict 178 data. The confusion matrix in Figure 4 shows that the DBN-ELM-BP model produces the most realistic positive predictive values. In contrast, the DBN model produces the most negative real values.

**Figure 5:**
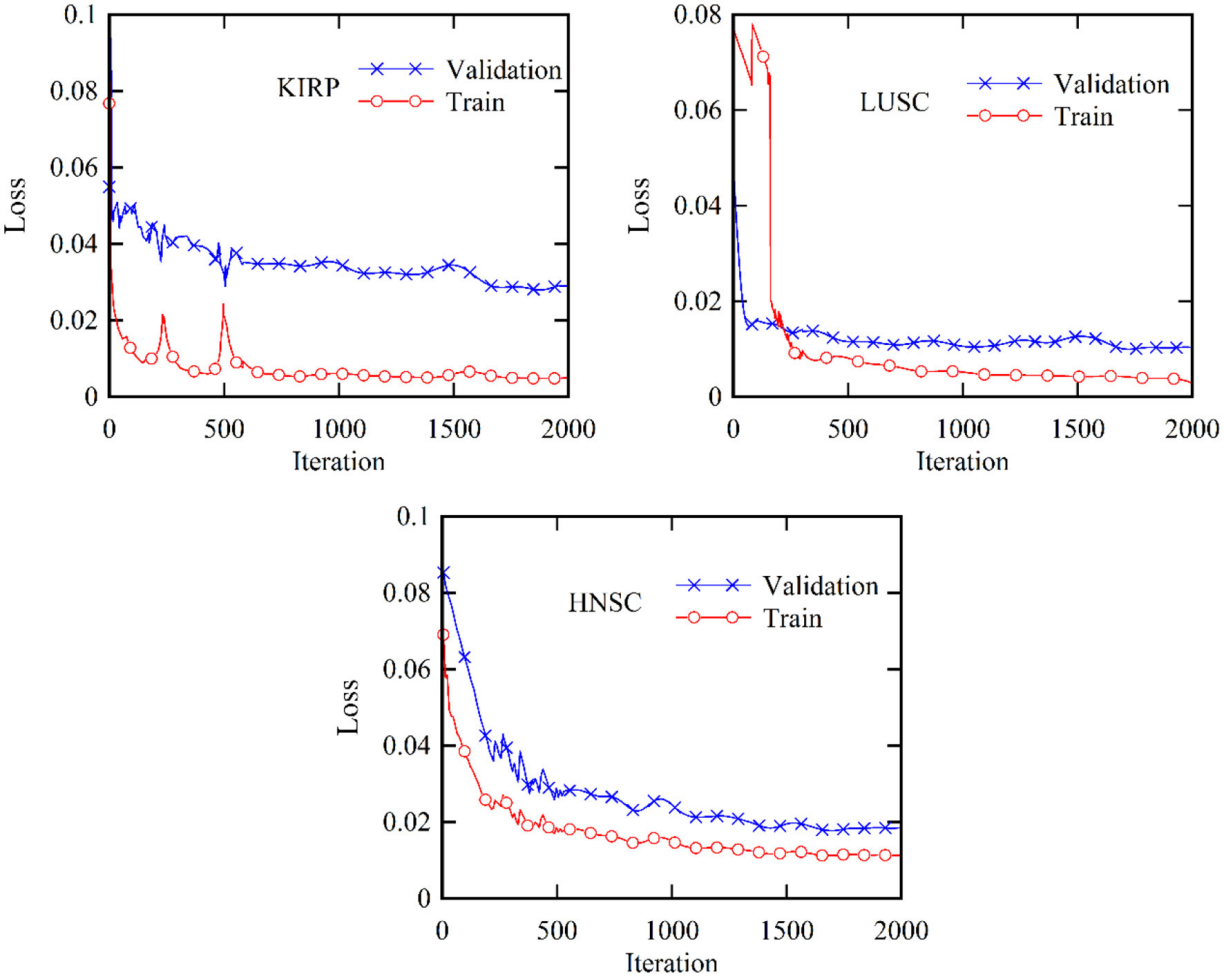
Validation and training loss profiles for the KIRP, LUSC, and HNSC datasets based on the proposed DBN-ELM-BP network for multi-omics data.

**Figure 6:**
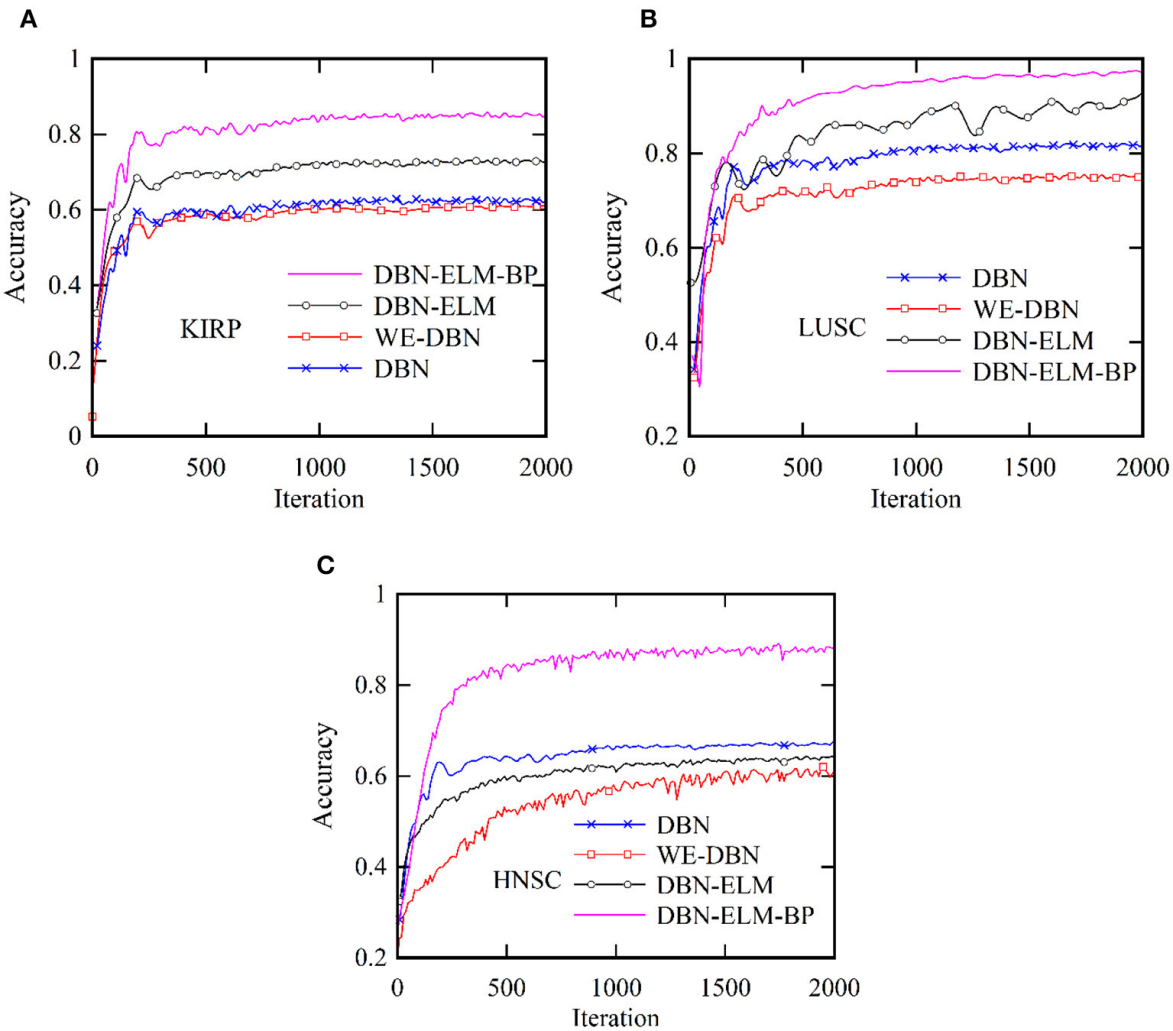
Comparative analysis of the accuracy of the proposed DBN-ELM-BP model for multi-omics data about other networks across three distinct datasets: **(A)** KIRC, **(B)** LUSC, and **(C)** HNSC.

Figure 5 provides a comprehensive visualization in the form of a loss diagram, presenting the dynamic behavior of the DBN-ELM-BP network across three distinct datasets: KIRP, LUSC, and HNSC. This representation encapsulates the interplay of the network during both the training and validation phases. Notably, the diagram reveals a convergence trend in the network's training, indicating a stabilization of the learning process, which becomes prominent around the 1,000th step. An interesting observation emerges as the loss on the validation data surpasses that of the training data, suggesting the potential generalization performance of the network in diverse scenarios. Turning attention to Figure 6, a detailed comparative analysis unfolds, showcasing the accuracy metrics of prominent networks—DBN, WE-DBN, DBN-ELM, and DBN-ELM-BP—across the three datasets: KIRP, LUSC, and HNSC. Remarkably, the hybrid DBN-ELM-BP network stands out with the highest accuracy across all three datasets. Specifically, the accuracy achieved with this algorithm for KIRP, LUSC, and HNSC datasets is reported as 88.31%, 91.42%, and 77.51%, respectively. These results underscore the superior performance and robustness of the DBN-ELM-BP hybrid network, positioning it as an effective and reliable model for the prediction tasks when compared to alternative models. This visual representation offers a clear assessment of the performance of the DBN-ELM-BP model relative to alternative networks for each specific dataset, providing valuable insights into its efficacy in different cancer-type predictions.

**Figure 7:**
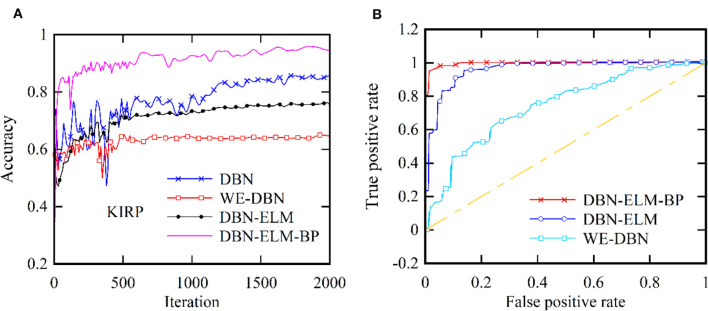
Two distinct aspects related to the KIRP datasets: **(A)** accuracy at each iteration and **(B)** the ROC curve.

Moreover, following the generation of model predictions for each class, the model's confidence level is computed by considering its accuracy, precision, and recall. As a class prediction model, the goal is to assess the trustworthiness of the accuracy percentage. Table 4 presents the outcomes concerning sensitivity, accuracy, and specificity obtained from the KIRC cancer dataset. Increased sensitivity and diagnostic specificity contribute to elevated accuracy and the area under the ROC curve, as depicted in Figure 7, which illustrates the accuracy and ROC curves for various neural network models. The accuracy plot (Figure 7A) offers insights into how accuracy evolves over different iterations, providing a dynamic view of the model's performance. On the other hand, the ROC curve (Figure 7B) illustrates the trade-off between sensitivity and specificity, offering a comprehensive evaluation of the model's prediction capabilities for the KIRP datasets. The outcomes of this study suggest that the DBN-ELM-BP model attains the highest accuracy and sensitivity levels. Additionally, the DNN model demonstrates favorable specificity in comparison to other models.

**Table 4:** Comparative overview of prediction accuracy among various prediction models utilizing the KIRC dataset.

**Methods**	**Performance measures**			
	**ACC (%)**	**SEN (%)**	**SPE (%)**	**F1-score (%)**
DBN	74.37	82.29	56.64	71.23
WE-DBN	70.76	79.34	45.43	75.67
DBN-ELM	87.38	92.26	52.72	80.34
DBN-ELM-BP	**94.61**	**94.73**	60.23	**86.56**
DNN	75.67	73.54	**67.60**	79.98
Google net	74.74	77.45	65.45	74.15

Bold values indicate the appropriate values in each column of the table.

Tables 5, 6 present an evaluation of the performance of various neural network methods on DNA methylation data about KIRP, LUSC, and HNSC cancers. Specifically, Table 5 provides a comparison of results between DBN, WE-DBN, DBN-ELM, DBN-ELM-BP, and sequential methods such as DNN and GoogleNet for the KIRP dataset. The outcomes highlight that the DBN-ELM-BP prediction method exhibits notable performance compared to other methods. Notably, the DBN-ELM-BP network attains the highest values for ACC, SEN, and F1-score, indicating superior overall performance. In contrast, DBN-ELM shows the least favorable values for ACC and SPE. Interestingly, WE-DBN stands out as the best-performing method based on the SPE criterion. For the KIRP dataset, the DBN-ELM-BP network achieves an ACC of 97.09%, SEN of 89.16%, SPE of 75.45%, and an F1-score of 86.45%. These results underscore the efficacy of the DBN-ELM-BP prediction method for the KIRP dataset.

**Table 5:** A comparative analysis of prediction accuracy across various prediction models using the KIRP dataset.

**Methods**	**Performance measures**			
	**ACC (%)**	**SEN (%)**	**SPE (%)**	**F1-score (%)**
DBN	88.22	78.06	63.41	74.60
WE-DBN	68.00	76.40	74.37	72.93
DBN-ELM	77.87	78.82	59.98	75.69
DBN-ELM-BP	**97.09**	**89.16**	70.72	**86.45**
DNN	78.08	73.78	**75.45**	77.08
Google net	75.66	75.78	72.43	75.21
ResNet	87.56	78.42	74.16	82.64

Bold values indicate the appropriate values in each column of the table.

**Table 6:** A comparative analysis of prediction accuracy across various prediction models using the LUSC dataset.

**Methods**	**Performance measures**			
	**ACC (%)**	**SEN (%)**	**SPE (%)**	**F1-score (%)**
DBN	86.72	81.90	66.21	49.22
WE-DBN	78.42	80.14	63.52	43.77
DBN-ELM	92.14	79.61	62.53	57.87
DBN-ELM-BP	**97.85**	**98.34**	77.85	68.16
DNN	83.45	77.33	78.23	65.38
GoogleNet	84.06	79.48	**80.79**	**69.10**
ResNet	89.96	82.13	77.72	67.91

Bold values indicate the appropriate values in each column of the table.

Table 6 provides the prediction results for the LUSC dataset. Notably, DBN-ELM-BP demonstrates superior accuracy and sensitivity, while GoogleNet exhibits the best performance in terms of specificity (SPE) and F1-score. WE-DBN records the lowest F1-score among the evaluated methods. The outcomes in Table 7, consistent with previous datasets, underscore the superior performance of the DBN-ELM-BP method based on accuracy (ACC) and sensitivity (SEN) for the HNSC dataset. In this case, GoogleNet and DNN excel in specificity (SPE) and F1-score criteria, respectively. For the LUSC dataset, the resulting accuracy, sensitivity, specificity, and F1-score values for the DBN-ELM-BP method are reported as 97.85%, 98.34%, 80.79%, and 69.10%, respectively. Also, for the HNSC dataset, the resulting accuracy, sensitivity, specificity, and F1-score values for the DBN-ELM-BP method are reported as 89.68%, 87.44%, 56.63%, and 73.70%, respectively. It's noteworthy that DBN-ELM-BP exhibits robustness and accuracy, particularly in scenarios with highly imbalanced datasets. Additionally, DNN demonstrates performance closely aligned with GoogleNet in predicting the cancer stage.

**Table 7:** A comparison of the prediction accuracy of different prediction models using the HNSC dataset.

**Methods**	**Performance measures**			
	**ACC (%)**	**SEN (%)**	**SPE (%)**	**F1-score (%)**
DBN	71.84	72.72	48.73	67.27
WE-DBN	62.16	67.64	47.06	65.29
DBN-ELM	64.87	76.62	43.77	66.74
DBN-ELM-BP	**89.68**	**87.44**	58.63	73.70
DNN	70.65	65.80	62.03	**81.67**
Google net	76.31	69.44	**69.37**	68.38
ResNet	69.05	62.36	53.20	72.63

Bold values indicate the appropriate values in each column of the table.

Tables 8–11 illustrate the impact of molecular data on predictive performance. It's essential to clarify that the primary goal of this section is not to assess the performance of various machine-learning algorithms through parameter modification. Instead, the objective is to evaluate how different molecular datasets influence the prediction of cancer stage, using the default parameters of DBN-ELM-BP. The DBN-ELM-BP approach, when applied to different molecular datasets as feature sets for stage prediction, demonstrated improved predictive performance. The maximum accuracy (ACC) scores across the four cancers were 95.34% for KIRC, 97.09% for KIRP, 97.58% for LUSC, and 89.68% for HNSC. Despite the ACC score achieved by the multi-omics dataset of KIRC being slightly lower than the standalone Methy dataset (84.49%), it showed greater significance in several other evaluation indicators such as ACC, SEN, SPE, and F1-score. This underscores the importance of integrating DNA methylation with mRNA expression, as combining these two types of molecular data can yield more accurate results for various stages of cancer. The results highlight the effectiveness of the DBN-ELM-BP approach in leveraging different molecular datasets for enhanced predictive performance in cancer stage prediction. The findings indicate that, for cancers KIRP, LUSC, and HNSC, leveraging multi-omics information yields the highest accuracy. Conversely, in the context of type KIRC cancer, employing Methy-data proves to be advantageous, particularly in predicting stages I and II of the disease.

**Table 8:** Predictive performances of DBN-ELM-BP algorithms for three molecular datasets of KIRC.

**KIRC**	**Performance measures**			
	**ACC (%)**	**SEN (%)**	**SPE (%)**	**F1-score (%)**
Methy	**95.34**	**96.09**	56.67	68.65
mRNA	97.56	91.87	53.45	73.46
Multi-omics	94.61	94.73	**60.23**	**86.56**

A bolded performance indicates a notable one.

**Table 9:** Predictive performances of DBN-ELM-BP algorithms for three molecular datasets of KIRP.

**KIRP**	**Performance measures**			
	**ACC (%)**	**SEN (%)**	**SPE (%)**	**F1-score (%)**
Methy	84.56	85.45	**87.43**	83.56
mRNA	62.34	82.53	74.22	77.42
Multi-omics	**97.09**	**89.16**	70.72	**86.45**

Bold values indicate the appropriate values in each column of the table.

**Table 10:** Predictive performances of DBN-ELM-BP algorithms for three molecular datasets of LUSC.

**LUSC**	**Performance measures**			
	**ACC (%)**	**SEN (%)**	**SPE (%)**	**F1-score (%)**
Methy	83.61	89.12	61.34	63.56
mRNA	75.23	93.23	59.34	65.34
Multi-omics	**97.85**	**98.34**	**77.85**	**68.16**

Bold values indicate the appropriate values in each column of the table.

**Table 11:** Predictive performances of DBN-ELM-BP algorithms for three molecular datasets of HNSC.

**HNSC**	**Performance measures**			
	**ACC (%)**	**SEN (%)**	**SPE (%)**	**F1-score (%)**
Methy	74.45	70.33	54.12	67.37
mRNA	69.45	79.23	**62.95**	62.65
Multi-omics	**89.68**	**87.44**	58.63	**73.70**

Bold values indicate the appropriate values in each column of the table.

## 4 Discussion

The discussion of the study involves a comparison of the proposed DBN-ELM-BP model with existing studies in the realm of determining the early- and late- stages of cancer. Rahimi and Gönen (2018) utilized a multiple kernel learning model and achieved an 86% prediction accuracy for KIRP cancer. In comparison, our proposed DBN-ELM-BP model attained a higher accuracy of 97.09%, indicating a notable 12.5% improvement.

Deng et al. (2016), who employed DNA methylation to predict KIRC stages, reported an accuracy of 0.696, which is approximately 35% lower than the results obtained from our current model. Bhalla et al. (2017) focused on using gene data to identify the early and late stages of KIRC, achieving a maximum accuracy of 72.6%. In contrast, the proposed research achieved a higher accuracy of 95.34%.

Additionally, the study by Ma et al. (2020), which introduced the new XGBoost method, reported accuracies of 0.719, 0.835, 0.783, and 0.837 for KIRC, KIRP, HNSC, and LUSC, respectively. In comparison, the proposed hybrid neural network outperformed these results with accuracies of 0.953, 0.971, 0.897, and 0.978 for the corresponding datasets.

These comparisons underscore the superior performance of the presented deep learning method in predicting the characteristics of different cancers with high accuracy. The potential for extending this method to other types of cancers and larger datasets is suggested for future research, highlighting the versatility and promising outcomes of the proposed approach.

## 5 Conclusion

The significance of timely and accurate cancer prediction and diagnosis necessitates the development of effective methods for disease identification. Various prediction techniques can be employed to predict cancer at different stages. In this study, we propose a novel framework based on diverse deep learning models, including DBN, WE-DBN, DBN-ELM, and DBN-ELM-BP, for the diagnosis of early- and late-stage cancers using gene sets. The framework aims to achieve high accuracy while maintaining low computational time. The key innovation lies in the utilization of a modified segmentation approach within the DBN model for the prediction process.

Compared to prior studies, our model showcases a notable 12% enhancement in predictive accuracy. Noteworthy is the fact that this improvement is realized through a considerably streamlined construction of DBN-ELM-BP. The methods we propose demonstrate a remarkably high diagnostic performance in predicting cancer stages, encompassing both early and late phases, as reflected in the results. These findings suggest the promising potential of our DBN-ELM-BP model to unveil cancer-specific markers tailored to each cancer type. This optimism stems from the prospect that continued refinements may pave the way for identifying markers conducive to early cancer detection.

## Data availability statement

Publicly available datasets were analyzed in this study. This data can be found here: Ma et al. (2020).

## Author contributions

AA: Investigation, Methodology, Software, Writing—review & editing. AC: Writing—original draft, Writing—review & editing.
